# Birkhoff Normal Forms and KAM Theory for Gumowski-Mira Equation

**DOI:** 10.1155/2014/819290

**Published:** 2014-01-16

**Authors:** M. R. S. Kulenović, Z. Nurkanović, E. Pilav

**Affiliations:** ^1^Department of Mathematics, University of Rhode Island, Kingston, RI 02881-0816, USA; ^2^Department of Mathematics, University of Tuzla, 75000 Tuzla, Bosnia and Herzegovina; ^3^Department of Mathematics, University of Sarajevo, 71000 Sarajevo, Bosnia and Herzegovina

## Abstract

By using the KAM theory we investigate the stability of equilibrium solutions of the Gumowski-Mira equation: *x*_*n*+1_ = (2*ax*_*n*_)/(1 + *x*_*n*_^2^) − *x*_*n*−1_, *n* = 0,1,…, where *x*_−1_, *x*_0_ ∈ (−∞, ∞), and we obtain the Birkhoff normal forms for this equation for different equilibrium solutions.

## 1. Introduction and Preliminaries

The Gumowski-Mira equation [1] is given by

(1) $$\begin{array}{c} \begin{array}{c} x_{n+1}=y_{n}+F\left(x_{n}\right),\\ y_{n+1}=-x_{n}+F\left(x_{n+1}\right), \end{array}\}\;\left(n=0,1,\ldots \right), \end{array}$$

where *F* is one of the functions

(2) $$\begin{array}{c} au+b\frac{u}{1+u},\;\;b\frac{u}{1+u^{2}},\;\;b\frac{u^{2}}{1+u^{2}},\\ \mu u+\left(1-\mu \right)x^{n},\;n=2,3,\;\;\mu \in \left(-1,1\right), \end{array}$$

and the parameters *a* and *b* are positive. These equations were considered by Gumowski and Mira in a series of papers and the book [1]. System (1) implies

(3) $$\begin{array}{c} x_{n+2}=y_{n+1}+F\left(x_{n+1}\right)=-x_{n}+2F\left(x_{n+1}\right),\;n=0,1,\ldots , \end{array}$$

and so {*x*_*n*_} satisfies the difference equation

(4) $$\begin{array}{c} x_{n+2}=2F\left(x_{n+1}\right)-x_{n},\;n=0,1,\ldots . \end{array}$$

In this paper we will consider (4) with *F*(*u*) = *au*/(1 + *u* ^2^), where *a* > 0 and the initial conditions are real numbers; that is, we consider

(5) $$\begin{array}{c} z_{n+1}=\frac{2az_{n}}{1+z_{n}^{2}}-z_{n-1},\;n=0,1,\ldots , \end{array}$$

where *a* > 0 and the initial conditions *z*_−1_ and *z*_0_ are real numbers.

Several authors have studied the Gumowski-Mira equation (5) and have obtained some results on the stability of the equilibrium points, the bifurcation of the global behavior of solutions, periodic and chaotic solutions, and so forth; see [1–11]. By using the methods of algebraic and projective geometry in [2, 3, 5, 6] the authors obtained very precise description of complicated global behavior which includes finding the feasible periods of all periodic solutions, proving the existence of chaotic solutions through conjugation of maps, and so forth. These methods were first used by Zeeman [12] for the study of so-called Lyness' equation. All these methods are based on the careful algebraic study of an invariant that (5) possesses. Our method here is purely analytic and is based on the asymptotic expansions. This method can be used to obtain similar results about periodic and chaotic solutions.

Our method is based on the application of KAM theory (Kolmogorov-Arnold-Moser), which brings the considered equation to certain normal form which, in addition to investigation of stability of an equilibrium, can be used to find different periodic solutions, chaotic solutions, and so forth. This technique was used successfully in [13–16] in the case of difference equations while there exists vast literature in the case of differential equations; see [17, 18]. Computer simulations of the trajectories of (5) indicate the existence of an infinite nested family of invariant closed curves surrounding an elliptic fixed point, sequences of periodic islands in the regions between the invariant curves, and stochastic regions surrounding the periodic islands and between invariant closed curves. Furthermore, the entire structure seems to exhibit self-similar structure.

The main feature of (5) is that the corresponding map is the area-preserving map of the plane having a nondegenerate elliptic fixed point. We show that the complicated orbit structure near the elliptic fixed point is an immediate consequence of classical results from the KAM theory. Away from the elliptic fixed point, the KAM theory does not apply and one has to study the geometric structures through some other analytical, algebraic, or geometric methods, such as those in [2, 3, 5, 6, 11, 12]. Most important geometric structures of interest for (5) are periodic points, which are typically of hyperbolic or elliptic type; the invariant manifolds associated with hyperbolic periodic points; KAM invariant curves (around the elliptic fixed point or around elliptic periodic orbits); and cantori, which are remnant sets of Cantor type of destroyed invariant circles. The building blocks for these structures are the periodic orbits, as the other geometric objects can be obtained as limits of periodic orbits.

First, we present the basic results that will be used in the sequel. See [10, 13, 17–19].

**Theorem 1 (Birkhoff normal form)**

Let **F** : ℝ^2^ → ℝ^2^ be an area-preserving *C* ^*n*^ map (*n*  times continuously differentiable) with a fixed point at the origin whose complex-conjugate eigenvalues *λ* and $\bar{\lambda }$ are on the unit disk (elliptic fixed point).

Suppose there exists an integer *l* such that

(6) $$\begin{array}{c} 4\leq l\leq n+1, \end{array}$$

and suppose that the eigenvalues satisfy

(7) $$\begin{array}{c} \lambda^{k}\neq 1\;for\;k=3,4,\ldots ,l. \end{array}$$

Let *r* = [*l*/2] be the integer part of *l*/2.

Then there exists a smooth function $g(z,\bar{z})$ that vanishes with its derivatives up to order *r* − 1 at *z* = 0, and there exists a real polynomial

(8) $$\begin{array}{c} \alpha \left(w\right)=\alpha_{1}w+\alpha_{2}w^{2}+\cdots +\alpha_{r}w^{r} \end{array}$$

such that the map **F** can be reduced to the normal form by suitable change of complex coordinates

(9) $$\begin{array}{c} z⟶F\left(z,\bar{z}\right)=\lambda ze^{i\alpha (z\bar{z})}+g\left(z,\bar{z}\right). \end{array}$$

In other words the corresponding system of difference equations

(10) $$\begin{array}{c} x_{n+1}=F\left(x_{n}\right) \end{array}$$

can be reduced to the form

(11) $$\begin{array}{c} \left[\begin{array}{c} r_{n+1}\\ s_{n+1} \end{array}\right]=\left[\begin{array}{cc} \cos\omega & -\sin\omega \\ \sin\omega & \cos\omega \end{array}\right]\left[\begin{array}{c} r_{n}\\ s_{n} \end{array}\right]+\left[\begin{array}{c} O_{l}\\ O_{l} \end{array}\right], \end{array}$$

where

(12) $$\begin{array}{c} \omega =\sum_{k=0}^{M}\gamma_{k}{\left(r_{n}^{2}+s_{n}^{2}\right)}^{k},\;M=\left[\frac{l}{2}\right]-1. \end{array}$$

Here *O*_*l*_ denotes a convergent power series in *r*_*n*_ and *s*_*n*_ with terms of order greater than or equal to *l* which vanishes at the origin and [*x*] denotes the least integer greater than or equal to *x*.

The numbers *γ*_1_,…, *γ*_*k*_ are called twist coefficients. Using Theorem 1 we can state the main stability result for an elliptic fixed point, known as the KAM theorem (or Kolmogorov-Arnold-Moser theorem); see [10, 18, 19].

**Theorem 2 (KAM theorem)**

Let **F** : ℝ^2^ → ℝ^2^ be an area-preserving map with an elliptic fixed point at the origin satisfying the conditions of Theorem 1. If the polynomial *α*(|*z*|^2^) is not identically zero, then the origin is a stable equilibrium point. In other words if for some *k* ∈ {1,…, *M*} one has *γ*_*k*_ ≠ 0 in (12), then the origin is a stable equilibrium point.

**Remark 3**

Consider an invariant annulus *A*_*ε*_ = {*z* : *ε* < |*z* | <2*ε*} in a neighbourhood of the elliptic fixed point, for *ε* a sufficiently small positive number. Note that the linear part of normal form approximation leaves all circles invariant. The motion restricted to each of these circles is a rotation by some angle. Also note that if at least one of the twist coefficients *γ*_*k*_ is nonzero, the angle of rotation will vary from circle to circle. A radial line through the fixed point will undergo twisting under the mapping. The KAM theorem says that, under the addition of the remainder term, most of these invariant circles will survive as invariant closed curves under the full map [17, 18]. Precisely, the following result holds see [13, 18].

**Theorem 4**

Assuming that $\alpha (z\overset{-}{z})$ is not identically zero and *ε* is sufficiently small, then the map *F* has a set of invariant closed curves of positive Lebesgue measure close to the original invariant circles. Moreover, the relative measure of the set of surviving invariant curves approaches full measure as *ε* approaches 0. The surviving invariant closed curves are filled with dense irrational orbits.

The following is a consequence of Moser's twist map theorem [18, 19].

**Theorem 5**

Let **F** : ℝ^2^ → ℝ^2^ be an area-preserving diffeomorphism and $\left(\overset{-}{x},\overset{-}{y}\right)$ a nondegenerate elliptic fixed point. There exist periodic points with arbitrarily large period in every neighbourhood of $\left(\overset{-}{x},\overset{-}{y}\right)$.

Indeed the theorem implies that arbitrarily close to the fixed point there are always infinitely many gaps between consecutive invariant curves that contain periodic points. Within these gaps, one finds, in general, orbits of hyperbolic and elliptic periodic points. These facts can hardly be seen from computer simulations since some periodic orbits can exist on very small scales.

The linearized part of (11) represents a rotation for angle *ω* and so if *ω* is rational multiple of *π* every solution is periodic with the same period while if *ω* is irrational multiple of *π* there will exist chaotic solutions. In this paper we will not go into detailed study of these behaviors.

## 2. Equilibrium Solutions and Linearized Stability Analysis

Equation (5) has at most three equilibrium points: $\overset{-}{y}=0$ for all values of parameter *a* and $\overset{-}{y}=\pm \sqrt{a-1}$ when *a* > 1. The linearized equation which corresponds to (5) at any equilibrium point $\overset{-}{y}$ is

(13) $$\begin{array}{c} z_{n+1}-2a\frac{1-{\overset{-}{y}}^{2}}{{\left(1+{\overset{-}{y}}^{2}\right)}^{2}}z_{n}+z_{n-1}=0, \end{array}$$

and its characteristic equation is

(14) $$\begin{array}{c} \lambda^{2}-2a\frac{1-{\overset{-}{y}}^{2}}{{\left(1+{\overset{-}{y}}^{2}\right)}^{2}}\lambda +1=0, \end{array}$$

which shows that the corresponding map is area preserving.

The characteristic equation at $\overset{-}{y}=0$ is

(15) $$\begin{array}{c} \lambda^{2}-2a\lambda +1=0, \end{array}$$

with characteristic roots $\lambda_{\pm }=a\pm \sqrt{a^{2}-1}$. When *a* > 1 characteristic roots are positive and *λ*_+_ > 1, *λ*_−_ ∈ (0,1) which shows that the zero equilibrium in the case *a* > 1 is a saddle point. In the case −1 < *a* < 1 characteristic roots are complex conjugate numbers lying on the unit circle, which means that the zero equilibrium is nonhyperbolic of the elliptic type; see [10, 19]. If *a* = 1 then the characteristic roots are both equal to 1 and the zero equilibrium is nonhyperbolic of the parabolic type. Similarly, if *a* = −1 then the characteristic roots are both equal to −1 and the zero equilibrium is nonhyperbolic of the parabolic type. In this paper we consider the case when *a* ≠ ±1.

If *a* < −1 then *λ*_±_ < 0 and we have $\left|\lambda_{+}\right|=\left|a+\sqrt{a^{2}-1}\right|<1$, $\left|\lambda_{-}\right|=\left|a-\sqrt{a^{2}-1}\right|>1$, which shows that the zero equilibrium is a saddle point.

The characteristic equation at $\overset{-}{y}=\pm \sqrt{a-1}$ is

(16) $$\begin{array}{c} \lambda^{2}-\frac{2\left(2-a\right)}{a}\lambda +1=0, \end{array}$$

with characteristic roots $\lambda_{\pm }=(2-a\pm 2\sqrt{1-a})/a$. When *a* > 1 characteristic roots are complex conjugate numbers lying on the unit circle, which means that the non-zero equilibrium solutions are nonhyperbolic of the elliptic type see [10, 19].

## 3. KAM Theory Applied to (5) at Zero Equilibrium for *a* ∈ (−1,1)∖{−(1/2), 0}

First, we use the substitution

(17) $$\begin{array}{c} z_{n}=y_{n},\\ z_{n+1}=x_{n}, \end{array}$$

to transform (5) to the system

(18) $$\begin{array}{c} \begin{array}{c} x_{n+1}=\frac{2ax_{n}}{1+x_{n}^{2}}-y_{n},\\ y_{n+1}=x_{n}, \end{array}\}\;n=0,1,\ldots . \end{array}$$

The corresponding linearized system at *E*_0_(0,0) is

(19) $$\begin{array}{c} \begin{array}{c} X_{n+1}=2aX_{n}-Y_{n},\\ Y_{n+1}=X_{n}, \end{array}\}\;n=0,1,\ldots . \end{array}$$

The characteristic equation of (19) is (15) with characteristic roots $\lambda_{\pm }=a\pm \sqrt{a^{2}-1}$. We consider the case where −1 < *a* < 1 in which case *E*_0_ is nonhyperbolic equilibrium of elliptic type.

A straightforward calculation gives the following expressions for second, third, and fourth power of the characteristic root:

(20) $$\begin{array}{c} \lambda^{2}={\left(a+i\sqrt{1-a^{2}}\right)}^{2}=2a^{2}-1+2ai\sqrt{1-a^{2}},\\ \lambda^{3}=a\left(4a^{2}-3\right)+\left(4a^{2}-1\right)i\sqrt{1-a^{2}},\\ \lambda^{4}=1+a\left(8a^{3}-8a+4\left(2a^{2}-1\right)i\sqrt{1-a^{2}}\right). \end{array}$$

Clearly, *λ* ^3^ ≠ 1 and *λ* ^4^ ≠ 1 for *a* ∈ (−1,1)∖{−(1/2), 0}. Thus the assumptions of Theorem 1 are satisfied for *l* = 4 and we will find the Birkhoff normal form of (18) by using the sequence of transformations described in Section 1.

### 3.1. First Transformation

Notice that the matrix of the linearized system (19) at the origin is given as

(21) $$\begin{array}{c} J_{0}=\left[\begin{array}{cc} 2a & -1\\ 1 & 0 \end{array}\right]. \end{array}$$

A straightforward calculation shows that the matrix of the corresponding eigenvectors which correspond to *λ* and $\bar{\lambda }$ of *J*_0_ is

(22) $$\begin{array}{c} P=\left[\begin{array}{cc} 1 & 1\\ \bar{\lambda } & \lambda \end{array}\right]. \end{array}$$

In order to obtain the Birkhoff normal form of system (18) we will expand the right-hand sides of the equations of system (18) at the equilibrium point (0,0) up to the order *l* − 1 = 3. We obtain

(23) $$\begin{array}{c} x_{n+1}=2ax_{n}-y_{n}-2ax_{n}^{3}+O_{4},\\ y_{n+1}=x_{n}. \end{array}$$

Now the change of variables

(24) $$\begin{array}{c} \left[\begin{array}{c} x_{n}\\ y_{n} \end{array}\right]=P\cdot \left[\begin{array}{c} u_{n}\\ v_{n} \end{array}\right]=\left[\begin{array}{c} u_{n}+v_{n}\\ \bar{\lambda }u_{n}+\lambda v_{n} \end{array}\right] \end{array}$$

or

(25) $$\begin{array}{c} x_{n}=u_{n}+v_{n},\\ y_{n}=\bar{\lambda }u_{n}+\lambda v_{n} \end{array}$$

transforms system (23) into

(26) un+1+vn+1=2a(un+vn)−(λ¯un+λvn)−2a(un+vn)3+O4,λ¯un+1+λvn+1=un+vn,

which after some simplifications becomes

(27) $$\begin{array}{c} u_{n+1}=\lambda u_{n}+\sigma {\left(u_{n}+v_{n}\right)}^{3}+O_{4},\\ v_{n+1}=\bar{\lambda }v_{n}+\bar{\sigma }{\left(u_{n}+v_{n}\right)}^{3}+O_{4}, \end{array}$$

where

(28) $$\begin{array}{c} \sigma =-\frac{2\lambda a}{\lambda -\bar{\lambda }}=a\left(-1+\frac{a}{\sqrt{1-a^{2}}}i\right),\\ \lambda^{2}-2a\lambda +1=0,\;\;\bar{\lambda }\lambda =1,\;\;\lambda +\bar{\lambda }=2a. \end{array}$$

### 3.2. Second Transformation

The objective of second transformation is to obtain the nonlinear terms up to order *l* − 1 in normal form. The change of variables

(29) un=ξn+ϕ2(ξn,ηn)+ϕ3(ξn,ηn),vn=ηn+ψ2(ξn,ηn)+ψ3(ξn,ηn),(n=0,1,…),

where

(30) $$\begin{array}{c} \phi_{k}\left(\xi ,\eta \right)=\overset{k}{\underset{j=0}{\sum }}a_{kj}\xi^{k-j}\eta^{j},\\ \psi_{k}\left(\xi ,\eta \right)=\overset{k}{\underset{j=0}{\sum }}{\bar{a}}_{kj}\xi^{j}\eta^{k-j}, \end{array}$$

for *k* = 2 and *k* = 3, yields

(31) un=ξn+(a20ξn2+a21ξnηn+a22ηn2)+(a30ξn3+a31ξn2ηn+a32ξnηn2+a33ηn3),vn=ηn+(a20¯ηn2+a21¯ξnηn+a22¯ξn2)+(a30¯ηn3+a31¯ξnηn2+a32¯ξn2ηn+a33¯ξn3),un2=ξn2+2a20ξn3+2a21ξn2ηn+2a22ξnηn2+O4,vn2=ηn2+2a20¯ηn3+2a21¯ξnηn2+2a22¯ξn2ηn+O4,un3=ξn3+O4,vn3=ηn3+O4,un2vn=ξn2ηn+O4,unvn2=ξnηn2+O4,unvn=a22¯ξn3+(a20+a21¯)ξn2ηn+(a21+a20¯)ξnηn2+ξnηn+a22ηn3+O4.

Solving these equations for *ξ*_*n*+1_ and *η*_*n*+1_ one obtain

(32) ξn+1=(λξn+α2ξn2ηn)+O4,ηn+1=(λ¯ηn+α¯2ξnηn2)+O4,n=0,1,….

By using (32) and $\bar{\lambda }=\left(2a-\lambda \right)$ in first equality of (31) and rescaling by replacing *n* with *n* + 1, we have

(33) un+1=λξn+a20λ2ξn2+a30λ3ξn3+a21ξnηn+(α2+a31λ)ξn2ηn+a32λ¯ξnηn2+a222λ¯2ηn2+a333λ¯3ηn3.

By using (33) in the left-hand side and (31) in the right-hand side of (27), we obtains

(34) λξn+a20λ2ξn2+a30λ3ξn3+a21ξnηn+(α2+a31λ)ξn2ηn  +a32λ¯ξnηn2+a222λ¯2ηn2+a333λ¯3ηn3 =λξn+λ(a20ξn2+a21ξnηn+a22ηn2)  +λ(a30ξn3+a31ξn2ηn+a32ξnηn2+a33ηn3)  +σ(ξn+ηn)3+O4.

The last relation holds if the corresponding coefficients are equal, which leads to the following set of equalities:

(35) ξn2:(λ2−λ)a20=0,ξn3:(λ3−λ−σ)a30=0,ξnηn:(1−λ)a21=0,ξn2ηn:α2−3σ=0.

Now

(36) $$\begin{array}{c} a_{20}=a_{21}=0,\\ \alpha_{2}=3\sigma =3a\left(-1+i\frac{a\sqrt{1-a^{2}}}{1-a^{2}}\right). \end{array}$$

This implies

(37) Re(α2)=−3a.

### 3.3. Third Transformation

The objective of the third transformation consists in expressing the terms in (32) as real values. This is achieved by using the transformation

(38) $$\begin{array}{c} \xi_{n}=r_{n}+is_{n},\\ \eta_{n}=r_{n}-is_{n}. \end{array}$$

Comparing the system obtained with (11) and using (12) for *l* = 4, we determine the twist coefficients *γ*_0_ and *γ*_1_.

We have

(39) cosγ0=Re(λ),  γ1=−1sinγ0·Re(α2).

In order to apply the KAM theorem we have to show that *γ*_1_ ≠ 0. Indeed

(40) $$\begin{array}{c} -1<\cos\gamma_{0}=a<1,\\ \sin\gamma_{0}=\pm \sqrt{1-a^{2}},\\ \gamma_{1}=\frac{-1}{\pm \sqrt{1-a^{2}}}\left(-3a\right)=\pm \frac{3a}{\sqrt{1-a^{2}}}. \end{array}$$

**Figure 1:**
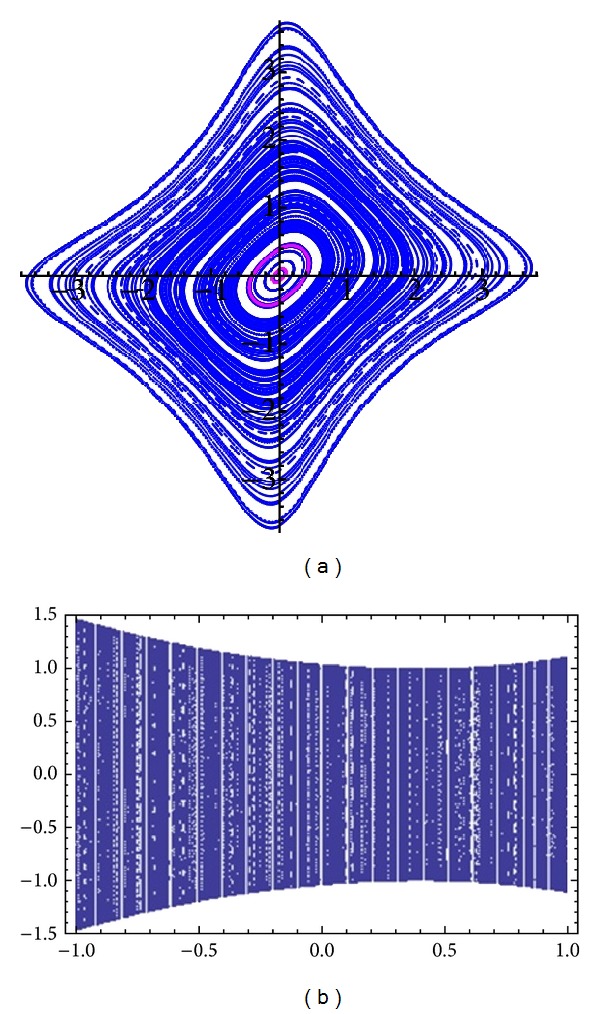
(a) A Phase portrait of three orbits for *a* = 0.5. (b) A bifurcation diagram of typical solution of (5) for *a* between −1 and 1. The plots are produced by *Dynamica* 3 [10].

Since *a* ∈ (−1,1)∖{−(1/2), 0}, this implies *γ*_1_ ≠ 0  (Figure 1).

Thus we have proved the following result.

**Theorem 6**

The zero equilibrium solution of (5) is stable for *a* ∈ (−1,1)∖{−(1/2), 0}.

## 4. KAM Theory Applied to (5) at Nonzero Equilibrium Solutions for *a* ∈ (1, +*∞*)∖{2,4}

In this section we will apply KAM theory to establish the stability of the non-zero equilibrium solutions for (5). First, we rescale (5) and then we use the substitution

(41) $$\begin{array}{c} \begin{array}{c} z_{n}=x_{n}+\sqrt{a-1},\\ y_{n}=x_{n-1}, \end{array}\}\;n=0,1,\ldots , \end{array}$$

to obtain the system

(42) $$\begin{array}{c} x_{n+1}=\frac{2a\left(x_{n}+\sqrt{a-1}\right)}{1+{\left(x_{n}+\sqrt{a-1}\right)}^{2}}-y_{n}-2\sqrt{a-1},\\ y_{n+1}=x_{n}, \end{array}$$

with the corresponding equilibrium point at the origin.

The linearized system of system (42) at (0,0) is

(43) $$\begin{array}{c} \begin{array}{c} X_{n+1}=\frac{2\left(2-a\right)}{a}X_{n}-Y_{n},\\ Y_{n+1}=X_{n}, \end{array}\}\;\left(n=0,1,\ldots \right) \end{array}$$

whose characteristic equation is (16) and the characteristic roots are $\lambda_{\pm }=\left(2-a\pm 2\sqrt{1-a}\right)/a$. As we mentioned earlier, when *a* > 1 the characteristic roots are complex conjugate numbers lying on the unit circle, which means that the non-zero equilibrium is nonhyperbolic of the elliptic type and so KAM theory is a natural tool to be applied.

A straightforward calculation gives the following expressions for second, third, and fourth power of the characteristic root:

(44) λ=2−a+2ia−1a,λ2=1a2(8−8a+a2+4i(2−a)a−1),λ3=1a3((2−a)(16−16a+a2)+2(16−16a+3a2)ia−1),λ3=1⟺a=4,λ4=1a4((8−8a+a2)2−16(2−a)2(a−1)+8i(8−8a+a2)(2−a)a−1),λ4=1⟺a=2.

It can be shown that *λ* ^3^ ≠ 1 and *λ* ^4^ ≠ 1 for *a* ∈ (1, +*∞*)∖{2,4} and so *l* = 4.

### 4.1. First Transformation

Notice that the matrix of the linearized system (43) is given as

(45) $$\begin{array}{c} J_{0}=\left[\begin{array}{cc} \frac{2\left(2-a\right)}{a} & -1\\ 1 & 0 \end{array}\right]. \end{array}$$

A straightforward calculation shows that the matrix of the corresponding eigenvectors which correspond to *λ* and $\bar{\lambda }$ of *J*_0_ is

(46) $$\begin{array}{c} P=\left[\begin{array}{cc} 1 & 1\\ \bar{\lambda } & \lambda \end{array}\right]. \end{array}$$

In order to obtain the Birkhoff normal form of system (42) we will expand the right hand sides of the equations of system (42) at the equilibrium point (0,0) up to the order *l* − 1 = 3. We obtain

(47) xn+1=−2(a−2)axn−yn+2(a−4)a−1a2×(xn2−(a2−8a+8)a(a−4)a−1xn3)+O4,yn+1=xn.

Next we use the change of variables

(48) $$\begin{array}{c} \left[\begin{array}{c} x_{n}\\ y_{n} \end{array}\right]=P\cdot \left[\begin{array}{c} u_{n}\\ v_{n} \end{array}\right]=\left[\begin{array}{c} u_{n}+v_{n}\\ \bar{\lambda }u_{n}+\lambda v_{n} \end{array}\right] \end{array}$$

or

(49) $$\begin{array}{c} x_{n}=u_{n}+v_{n},\\ y_{n}=\bar{\lambda }u_{n}+\bar{\lambda }v_{n}, \end{array}$$

and after tedious simplification we obtain the transformed system up to the terms of order three in the form

(50) $$\begin{array}{c} u_{n+1}=\lambda u_{n}+\sigma \left({\left(u_{n}+v_{n}\right)}^{2}+\sigma_{1}{\left(u_{n}+v_{n}\right)}^{3}\right),\\ v_{n+1}=\bar{\lambda }v_{n}+\bar{\sigma }\left({\left(u_{n}+v_{n}\right)}^{2}+\sigma_{1}{\left(u_{n}+v_{n}\right)}^{3}\right), \end{array}$$

where

(51) $$\begin{array}{c} \sigma =\frac{\lambda }{\lambda -\bar{\lambda }}\cdot \frac{2\left(a-4\right)\sqrt{a-1}}{a^{2}},\;\;\sigma_{1}=-\frac{\left(a^{2}-8a+8\right)}{a\left(a-4\right)\sqrt{a-1}}. \end{array}$$

### 4.2. Second Transformation

Similarly, as in the case of the zero equilibrium, we obtain

(52) a20=σλ2−λ=2(a−4)a−1a2(λ−1)(λ−λ¯),a21=−2σλ−1=(a−4)(a−1+i(a−1))2a(a−1),a22=σλ¯2−λ,2(a20+a22¯)=2(a−4)a−1a21λ−λ¯(2λ−1−2λ3−1),2(a20+a22¯)=(2(a−4)a−1a2)(−ai4a−1)×(2iaa−1(a−1)(a−4))=1a−1,α2=σ(4Re(a21)+2(a20+a22¯)+3σ1).

Now we simplify the right-hand sides of these expressions because the coefficient *α*_2_ plays the crucial role in determining stability of the equilibrium. We have

(53) 4Re(a21)+2(a20+a22¯)+3σ1 =2(a−4)a−1a(a−1)+1a−1−3(a2−8a+8)a(a−4)a−1 =4(a+2)aa−1(a−4),α2=((2−a)i−2a−1−4a−1)(2(a−4)a−1a2)×(4(a+2)aa−1(a−4))=2(a+2)(2a−1+i(a−2))a3a−1.

The real part of last expression is

(54) Re(α2)=4(a+2)a3.

### 4.3. Third Transformation

Using (39) we have

(55) $$\begin{array}{c} -1<\cos\gamma_{0}=\frac{2-a}{a}<1,\\ \sin\gamma_{0}=\pm \sqrt{1-{\left(\frac{2-a}{a}\right)}^{2}}=\pm \frac{2}{a}\sqrt{a-1},\\ \gamma_{1}=\pm \frac{2\left(a+2\right)}{a^{2}\sqrt{a-1}}. \end{array}$$

So, we have that *γ*_1_ ≠ 0 for all *a* ∈ (1, +*∞*)∖{2,4}.

The computation for the other non-zero equilibrium solution $-\sqrt{a-1}$ is similar.

Thus we have proved the following result.

**Theorem 7**

The equilibrium solution ${\overset{-}{y}}_{\pm }=\pm \sqrt{a-1}$ of (5) is stable for *a* ∈ (1, +*∞*)∖{2,4}.

**Remark 8**

**Figure 2:**
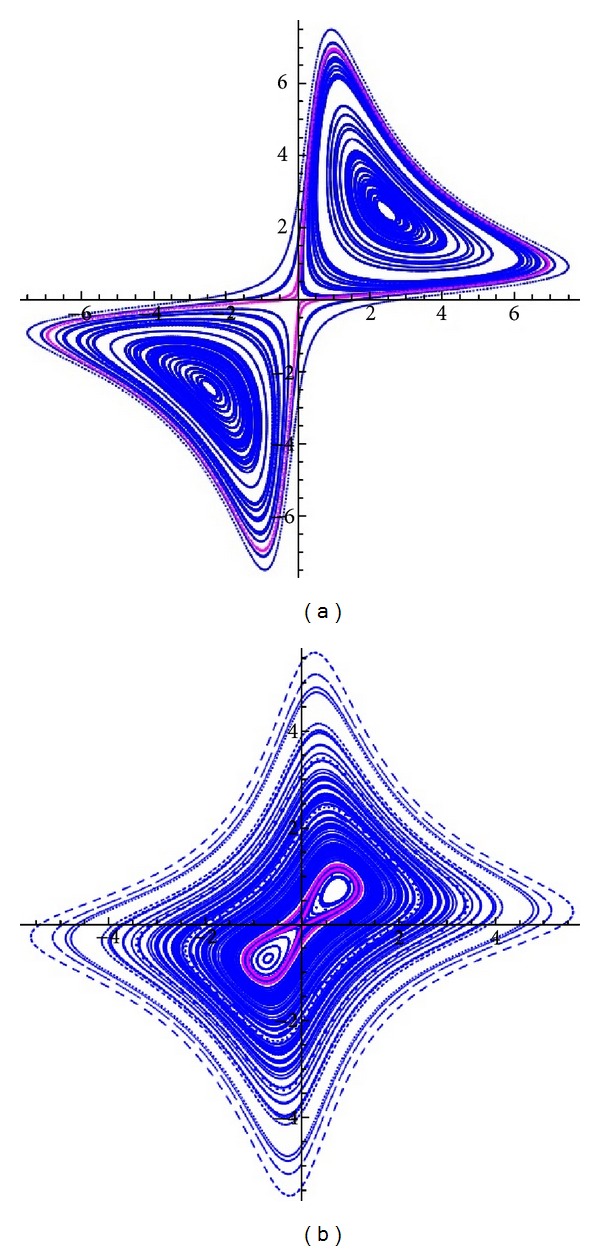
(a) A phase portrait of three orbits for *a* = 1.5. (b) A phase portrait of three orbits for *a* = 7. The plots are produced by *Dynamica* 3 [10].

**Figure 3:**
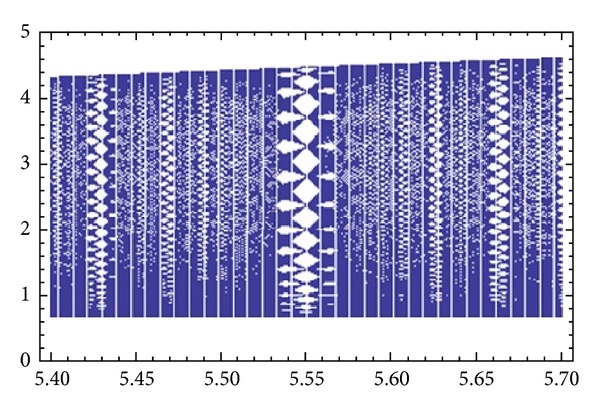
A bifurcation diagram in the (*a*, *x*)-plane for *x*_−1_ = 1.2 and *x*_0_ = 1.0 with parameter *a* in the range from 5.4 to 5.7. The plot is produced by *Dynamica* 3 [10].

The cases *a* = 2 and *a* = 4 can be treated by the method of local Lyapunov function as in [6, 9, 10, 20]. To do so one needs the corresponding invariant

(56) $$\begin{array}{c} I\left(x_{n},x_{n-1}\right)=x_{n}^{2}x_{n-1}^{2}+x_{n}^{2}+x_{n-1}^{2}-ax_{n}x_{n-1}, \end{array}$$

which assumes a minimum value at the isolated equilibrium point $\left(\overset{-}{y},\overset{-}{y}\right)$, and by Morse's lemma, see [19, 20], the level sets *I*(*x*, *y*) = *C* are diffeomorphic to the circles in the neighborhood of $\left(\overset{-}{y},\overset{-}{y}\right)$. This method can be extended further to give some global results on the dynamics of (5) as it was done in [6, 21] (Figures 2 and 3).

**Remark 9**

Theorems 6 and 7 show that (5) undergoes a bifurcation as the parameter *a* passes through 1. Precisely, as the parameter *a* passes through 1 the zero equilibrium changes its local character from a non-hyperbolic equilibrium point of elliptic type, when −1 < *a* < 1, to a saddle point, when *a* ∈ (−*∞*, −1) ∪ (1, +*∞*), where, at the critical value *a* = ±1, zero equilibrium is a non-hyperbolic equilibrium point of parabolic type. The positive equilibrium solutions $\pm \sqrt{a-1}$ are always the non-hyperbolic equilibrium points of elliptic type. The global change of behavior is that zero equilibrium loses its stability as the parameter *a* passes through ±1 and its stability is picked up by the positive equilibrium. So this bifurcation can be described as the exchange of stability bifurcation. The remaining case is dynamics at *a* = ±1 in which case the zero equilibrium, which is unique, is a non-hyperbolic equilibrium point of parabolic type. Finally, if *a* = 0 (5) reduces to

(57) $$\begin{array}{c} z_{n+1}=-z_{n-1},\;n=0,1,\ldots , \end{array}$$

which has the unique zero equilibrium and all solutions are periodic of period two in which case we have some trivial stability of equilibrium.

## References

[1] Gumowski I, Mira C (1980). *Recurrences and Discrete Dynamic Systems*.

[2] Bastien G, Rogalski M (2005). On the algebraic difference equations *u*
_*n*+2_
*u*
_*n*_ = *ψ*(*u*
_*n*+1_) in ℝ_∗_
^+^, related to a family of elliptic quartics in the plane. *Advances in Difference Equations*.

[3] Bastien G, Rogalski M (2007). On the algebraic difference equations *u*
_*n*+2_ + *u*
_*n*_ = *ψ*(*u*
_*n*+1_) in ℝ, related to a family of elliptic quartics in the plane. *Journal of Mathematical Analysis and Applications*.

[4] Beukers F, Cushman R (1998). Zeeman’s monotonicity conjecture. *Journal of Differential Equations*.

[5] Cima A, Gasull A, Maňosa V (2006). Dynamics of rational discrete dynamical systems via first integrals. *International Journal of Bifurcation and Chaos in Applied Sciences and Engineering*.

[6] Cima A, Gasull A, Maňosa V (2012). Non-autonomous two-periodic Gumowski-Mira difference equation. *International Journal of Bifurcation and Chaos in Applied Sciences and Engineering*.

[7] Clark CA, Janowski EJ, Kulenović MRS (2005). Stability of the Gumowski-Mira equation with period-two coefficient. *Journal of Mathematical Analysis and Applications*.

[8] Janowski EJ, Kulenović MRS, Nurkanović Z (2007). Stability of the kTH order lyness’ equation with a period-k coefficient. *International Journal of Bifurcation and Chaos*.

[9] Kulenović MRS (2000). Invariants and related Liapunov functions for difference equations. *Applied Mathematics Letters*.

[10] Kulenović MRS, Merino O (2002). *Discrete Dynamical Systems and Difference Equations with Mathematica*.

[11] MacKay RS (1993). *Renormalization in Area-Preserving Maps*.

[12] Zeeman EC (1996). *Geometric Unfolding of A Difference Equation*.

[13] Gidea M, Meiss JD, James, Ugarcovici I, Weiss H (2011). Applications of KAM theory to population dynamics. *Journal of Biological Dynamics*.

[14] Kocic VL, Ladas G, Tzanetopoulos G, Thomas E (1995). On the stability of Lyness' equation. *Dynamics of Continuous, Discrete and Impulsive Systems*.

[15] Kulenović MRS, Nurkanović Z (2008). Stability of Lyness' equation with period-two coeffcient via KAM theory. *Journal of Concrete and Applicable Mathematics*.

[16] Ladas G, Tzanetopoulos G, Tovbis A (1996). On May's host parasitoid model. *Journal of Difference Equations and Applications*.

[17] Tabor M (1989). *Chaos and Integrability in Nonlinear Dynamics. An Introduction*.

[18] Siegel C, Moser J (1971). *Lectures on Celestial Mechanics*.

[19] Hale JK, Kocak H (1991). *Dynamics and Bifurcation*.

[20] Bastien G, Rogalski M (2012). Level sets lemmas and unicity of critical points of invariants, tools for local stability and topological properties of dynamical systems. *Sarajevo Journal of Mathematics*.

[21] Duistermaat J (2010). *Discrete Integrable Systems. QRT Maps and Elliptic Surfaces*.

